# Conditional Reduction of Adult Born Doublecortin-Positive Neurons Reversibly Impairs Selective Behaviors

**DOI:** 10.3389/fnbeh.2015.00302

**Published:** 2015-11-12

**Authors:** Lillian Garrett, Jingzhong Zhang, Annemarie Zimprich, Kristina M. Niedermeier, Helmut Fuchs, Valerie Gailus-Durner, Martin Hrabě de Angelis, Daniela Vogt Weisenhorn, Wolfgang Wurst, Sabine M. Hölter

**Affiliations:** ^1^Institute of Developmental Genetics, Helmholtz Zentrum München, German Research Center for Environmental HealthNeuherberg, Germany; ^2^German Mouse Clinic, Helmholtz Zentrum München, German Research Center for Environmental HealthNeuherberg, Germany; ^3^Max Delbrück Zentrum für Molekulare MedizinBerlin, Germany; ^4^Technische Universität MünchenFreising-Weihenstephan, Germany; ^5^Deutsches Zentrum für Neurodegenerative Erkrankungen e. V. (DZNE)Munich, Germany; ^6^Munich Cluster for Systems Neurology (SyNergy), Ludwig-Maximilians-Universität MünchenMunich, Germany

**Keywords:** neurogenesis, doublecortin, social discrimination, emotionality, mice

## Abstract

Adult neurogenesis occurs in the adult mammalian subventricular zone (SVZ) along the walls of the lateral ventricles and the subgranular zone (SGZ) of the hippocampal dentate gyrus. While a burgeoning body of research implicates adult neurogenesis in olfactory bulb (OB)- and hippocampal-related behaviors, the precise function continues to elude. To further assess the behavioral importance of adult neurogenesis, we herein generated a novel inducible transgenic mouse model of adult neurogenesis reduction where mice with CreER^T2^ under doublecortin (DCX) promoter control were crossed with mice where diphtheria toxin A (DTA) was driven by the Rosa26 promoter. Activation of DTA, through the administration of tamoxifen (TAM), results in a specific reduction of DCX+ immature neurons in both the hippocampal dentate gyrus and OB. We show that the decrease of DCX+ cells causes impaired social discrimination ability in both young adult (from 3 months) and middle aged (from 10 months) mice. Furthermore, these animals showed an age-independent altered coping behavior in the Forced Swim Test without clear changes in anxiety-related behavior. Notably, these behavior changes were reversible on repopulating the neurogenic zones with DCX+ cells on cessation of the TAM treatment, demonstrating the specificity of this effect. Overall, these results support the notion that adult neurogenesis plays a role in social memory and in stress coping but not necessarily in anxiety-related behavior.

## Introduction

Adult neurogenesis, the birth and assimilation of new neurons, occurs in two regions of the adult brain: the subgranular zone (SGZ) of the hippocampal dentate gyrus and the subventricular zone (SVZ) along the walls of the lateral ventricles. Through a multi-stage process, a proportion of the newly born cells from the SGZ migrate the short distance to be incorporated as granule cells into the hippocampal dentate gyrus. Analogously, cells emanate from the SVZ along a rostral migratory stream (RMS) to ultimately integrate into the olfactory bulbs (OBs) as interneurons (Lledo et al., 2006; Braun and Jessberger, 2014). Their functional integration into the existing hippocampal and OB circuitry takes place approximately 3–6 weeks after their birth. Despite the burgeoning body of literature available on these processes, the precise contribution of adult neurogenesis to overall brain function continues to elude.

Ionizing radiation and systemic administration of anti-mitotic drugs, as well as aging as a natural means to decrease neurogenesis, were applied with varied results (Aimone et al., 2011; Feierstein, 2012; Cameron and Glover, 2015). While important information was gleaned from such studies, they were curtailed by innumerable off-target effects like inflammatory changes and effects on other systems. Genetic models of manipulating neurogenesis offered the potential for enhanced specificity and refinement, indicating a role for adult neurogenesis in hippocampal-dependent functions including spatial learning (Deng et al., 2009; Vukovic et al., 2013), synaptic plasticity (Massa et al., 2011), pattern separation (Sahay et al., 2011), emotion-related behavior (Revest et al., 2009), and in olfactory-related mating and maternal behaviors, but not simple odor discrimination or retention of odor-associated memory (Imayoshi et al., 2008; Lazarini and Lledo, 2011; Sakamoto et al., 2011). Nevertheless, contradictory results, particularly concerning the role of neurogenesis in emotionality (Petrik et al., 2012), highlight problems that can also be associated with the genetic approach. This includes the possibility of influencing non-neuronal cell lineages (through targeting GFAP; Saxe et al., 2006) and Nestin (Deng et al., 2009; Singer et al., 2009) with the consequent necessity to develop ever-more complex genetic constructs and elaborate mouse line crossing to increase specificity (Imayoshi et al., 2011; Sakamoto et al., 2011, 2014).

We herein report the generation and characterization of a transgenic mouse model (DCXCreER^T2^; DTA) that permits the inducible and reversible reduction of newly born neurons in neurogenic zones, with minimum interference in other brain areas and peripheral systems. Doublecortin (DCX) is a microtubule-associated protein involved in neuronal migration during development and adulthood. DCX expression is transient during adult neurogenesis (approx. 30 days), tapering off with the appearance of mature neuronal markers largely confined to areas of continuous neurogenesis and rarely outside (Brown et al., 2003; Keays, 2007; von Bohlen und Halbach, 2011). Importantly, relative to other markers (Nestin, GFAP), DCX is particular to the neuronal lineage. Thus, in a previously developed mouse line for neuronal lineage fate mapping, where a tamoxifen (TAM)-inducible Cre recombinase (CreER^T2^) was expressed under the control of the DCX promoter (Zhang et al., 2010), CreER^T2^ was found in cells that were neuronally committed and not involved in gliogenesis. Here, we crossed these mice with a transgenic diphtheria toxin A (DTA) mouse line (R26:lacZbpAfloxDTA), where a *lacZ*bpA-flox DTA cassette was incorporated into the ubiquitously expressed ROSA26 promoter. Administration of TAM to these mice results in specific expression of the cell death-inducing gene DTA in DCX+ newly born neurons with their consequent ablation as long as TAM is present in the system. We analyzed multiple aspects of the behavior of these mice during a period where they had access to a TAM-rich food and the DTA transgene was thus activated. This included behaviors associated with both the hippocampus and the OBs, and we determined whether the age of the animal (3 or 10 months) influences the effect neurogenesis reduction has on behavior. We also assessed the behavior of these mice subsequent to removal of the TAM-enriched food. The absence of TAM from the system would halt DTA transgene expression and allow the repopulation of the neurogenic zones with DCX+ cells. This would then act as a control condition to show that functional changes were due to decreased neurogenesis and not due to permanent loss of non-proliferating DCX+ cells outside the neurogenic zones or to non-specific expression of Cre or the DTA transgene in other brain regions.

## Materials and Methods

### Animals

#### DCXCreER^T2^ Mice

Generation of the DCXCreER^T2^ mice has been described in detail previously (Zhang et al., 2010). In brief, a targeting plasmid was generated by subcloning a 2380-bp Sal I-Not I fragment of pCAG-CreER^T2^-bpA-SS1 vector containing the CreER^T2^ cDNA into the BamH I and Not I site of the phuDCX-3509-DsRed2 cassette, which contains the promoter region of human DCX, resulting in the phuDCX-3509-CreER^T2^. A 7.7-kb DCX-3’UTR (3’UTR) was amplified with RT-PCR, following the manufacturer’s instructions (Invitrogen Kit; catalog No. 11904-018). The Spe I and Not I sites were inserted in the 5’ terminal of primers respectively. PCR products were cloned into a pCRII vector (TOPO TA Cloning Kit; Invitrogen; catalog No. K4600-01) to obtain the pCRIITOPO-3’UTR plasmid. A 7.7-kb Spe I-Not I fragment of pCRII-TOPO-3’UTR was subcloned into the Spe I and Not I site of the phuDCX-3509-CreER^T2^ cassette to get the phuDCX-3509-CreER^T2^-3’UTR targeting plasmid. The targeting plasmid, phuDCX-3509-CreER^T2^-3’-UTR, was linearized by digestion with Sal I-Not I. The purified linearized DNA was microinjected into the pronuclei of fertilized oocytes of FVB inbred mice.

#### R26:lacZbpA/DTA^+/−^ Mice

Generation of the R26:lacZbpA/DTA^+/−^ mice (Brockschnieder et al., 2006) have been described previously. In brief, a construct consisting of a splice acceptor sequence from plasmid pSAβgeo, a lacZflox–DT-A cassette, and a PGK-Neo resistance cassette, in both orientations, was inserted into a unique XbaI site of pROSA26-1. A bovine growth hormone polyadenylation signal (bpA) was cloned into a HindIII restriction site between the 30-end of the lacZ orf and the downstream loxP site. The Sac II linearized targeting vector was introduced by electroporation into E14.1 embryonic stem (ES) cells. A targeted ES cell clone was injected into C57BL/6 blastocysts and generated germline chimeras.

**Figure 1:**
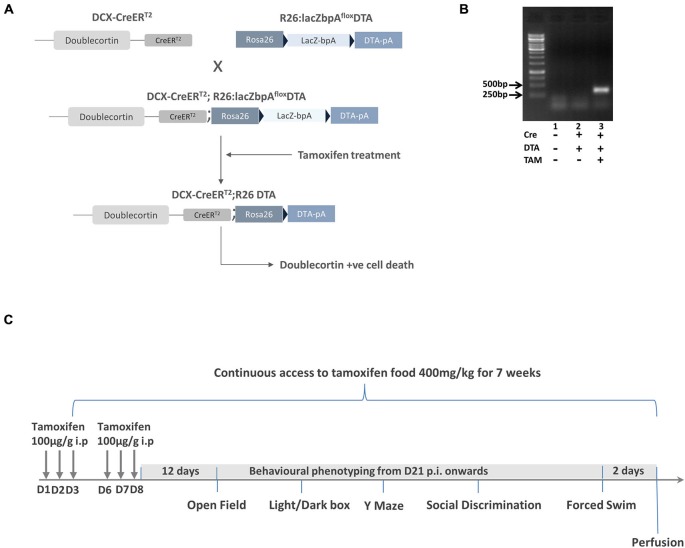
**Generation and characterization of DCXCreER^T2^; diphtheria toxin A (DTA) mice. (A)** Schematic showing the crossing of DCXCreER^T2^ mice with R26lacZbpa^flox^DTA mice to produce the DCXCreER^T2^; DTA double transgenic mice. The black arrow heads signify the position of the loxP sites. All mice were treated with tamoxifen. **(B)** Result of RT-PCR analysis showing positive band for DTA in the DCXCreER^T2^; DTA mice treated with TAM. *N* = 2–3 per group. **(C)** The experimental scheme for Experiment 1. p.i. post first TAM injection, D = day.

To expand the DCXCreER^T2^ and R26:lacZbpA/DTA^+/−^ transgenic mouse lines, they were backcrossed with wildtype C57BL/6J mice. DCX-CreER^T2+^ mice were then bred with R26:lacZbpA/DTA^+/−^ mice (Brockschnieder et al., 2006) resulting in DCX-CreER^T2+^; R26lacZbpAfloxDTA^+/−^ and DCX-CreER^T2+^; R26lacZbpAfloxDTA^+^ offspring (Figure 1A). In the R26:lacZbpA/DTA^+/−^ mice, the DTA transgene is under the control of the ubiquitous Rosa26 locus promoter, but expression is dependent on the Cre recombinase removal of a transcriptional STOP cassette. It is essential to note that the addition of a polyadenylation site (bpA) to these animals prevents the ectopic expression of the transgene and the potential degenerative effects that this incurs (Brockschnieder et al., 2006). We compared double transgenic mice (DCX-CreER^T2+^; R26lacZbpAfloxDTA^+^) with control, single transgenic littermate mice (DCX-CreER^T2+^; R26lacZbpAfloxDTA^−^). Both control and mutant mice were treated with TAM, ensuring that the group effects were not due to non-specific effects of this drug.

To assess the effect of age, we activated the DTA transgene expression with TAM administration at two different time points: when the animals were 3 (young adult) or 10 (middle age) months of age. Animals were housed in individually ventilated cages in a temperature (22–24°C) and humidity (50–60%) controlled environment on a 12/12 h light/dark cycle (lights on at 7 am). Each cage contained a mouse igloo. Water and food were available ad libitum. Both male and female offspring were used in the following experiments. All animal testing procedures were executed during the light phase of the cycle and approved by the German animal welfare authorities (Regierung von Oberbayern) and in accordance with the European Union Directive of the 22 September 2010 (2010/63/EU).

### Tamoxifen Treatment to Activate the Transgene

TAM (T-5648, Sigma-Aldrich) was dissolved in corn oil (C-8267, Sigma-Aldrich) at a stock concentration of 10 mg/ml. The mice received two rounds of treatment. In each round, the mice received 100μg TAM/g bodyweight (i.p.) once a day for three consecutive days. The rounds were separated by 2 days during which time animals had access to a special TAM diet (400 mg/kg, LASCRdiet^™^ CreActive TAM400, Lasvendi, Germany). The animals had continuous access to the TAM diet throughout the duration of the experiment unless indicated otherwise (during the reversal experiment 2).

### DTA Expression

To assess the expression of DTA in the mouse brain, mice with either the genotype CreER^T2^: DTA without tamoxifen (*n* = 2), CreER^T2^: DTA with tamoxifen (*n* = 2; 50 mg/kg i.p. once per day for 5 consecutive days) or wild type C57BL/6J (*n* = 3), were euthanized 2 days after the last tamoxifen treatment and the hippocampus was dissected out. Total RNA was isolated from adult hippocampus with the RNeasy Mini Kit (Qiagen/Germany) following the manufacturer’s protocol. One μg total RNA was reverse transcribed using the SuperScript^®^ One-Step RT-PCR System (Invitrogen/USA) according to the manufacturer’s instructions. DTA fragments were amplified by PCR (polymerase chain reaction) using the DTA forward primer (CGACAATAAATACGACGCTGCGGG) and DTA reverse primer (CATCGCATCTTGGCCACGTTTTCC). The annealing temperature was 56°C, the expected PCR size was 367 bp and the number of cycles was 35.

### Behavior Analysis

For Experiment 1, to assess the effects of activating DTA transgene expression in 3 and 10 month old mice, 21 days after the first TAM injection (i.e., the “time of induction”), the mice underwent a series of behavior tests according to the timeline and sequence shown in Figure 1C. For Experiment 2 (timeline shown in Figure 5A), to assess the effects of reversing the DTA transgene activation after an initial induction with TAM, the mice were tested in an identical behavior sequence. On this occasion, however, the transgene was first activated as described in Experiment 1 and the mice had continuous access to TAM-enriched food for 7 weeks during which time they remained experimentally naïve. The TAM-enriched food was subsequently removed and the mice were given a 6 week washout period where they had access to standard laboratory chow. Behavior testing then began in a sequence identical to that shown in Experiment 1 (Open Field, Light/Dark Box, Y Maze, Social Discrimination, Forced Swim Test). In both Experiment 1 and Experiment 2, all mice were sacrificed 2 days after the Forced Swim Test. Mice in Experiment 2 were aged 9 months at the start of behavior testing. In the interests of ensuring reproducibility, in a subset of mice from Experiment 2 (*n* = 6 controls, *n* = 6 transgenic) we performed the behavioral testing sequence as described in Experiment 1, while the transgene was activated.

### Open Field

The Open Field analysis was carried out as we described previously (Garrett et al., 2012; Hölter et al., 2013; Zimprich et al., 2014). It consisted of a transparent and infra-red light permeable acrylic test arena with a smooth floor (internal measurements: 45.5 × 45.5 × 39.5 cm). Illumination levels were set at approx. 150 lux in the corners and 200 lux in the middle of the test arena. Data were recorded and analyzed using the ActiMot system (TSE, Bad Homburg, Germany).

### Light/Dark Box

The test box was made of Plexiglas and divided into two compartments, connected by a small tunnel (4.5 × 5.6 × 13 cm high). The lit compartment (26.1 × 22.6 × 26 cm high) was made of transparent Plexiglas and was illuminated by cold light with an intensity of 650 lux in the middle; the dark compartment (14 × 22.6 × 26 cm high) was opaque, with a lid and not directly illuminated (approx. 5 lux in the center). The mouse was placed in the center of the dark compartment facing the hind wall and allowed to freely explore the apparatus for 5 min. Data were recorded and analyzed using the ActiMot infrared beam break system (TSE, Bad Homburg, Germany).

### Y Maze

Spontaneous alternations were assessed using the Y Maze, which was made of opaque light gray PVC and had three identical arms (30 × 5 × 15 cm) placed at 120° from each other; illumination in the center of the maze was 100 lux (Wall et al., 2003). Each mouse was placed at the end of one arm and allowed to move freely through the maze during a 5 min session. Spontaneous alternations (defined as consecutive entries into all three arms without repetitions in overlapping triplet sets) were scored. Total numbers of arm entries were collected cumulatively over the 5 min. Spontaneous alternation performance percentage is defined as the ratio of actual (total alternations) to possible alternations (total number of triplets) × 100. When placed in the Y Maze, normal mice prefer to explore the least recently visited arm, and thus tend to alternate visits between the three arms. To explore the three arms successively the mouse must maintain an ongoing record of the most recently visited arms, and continuously update such records. Therefore alternation behavior is a measure of spatial working memory.

### Forced Swim Test

The Forced Swim Test was carried out as described previously with adjustments (Deussing et al., 2010). The apparatus was a 10 L glass cylinder (24.5 cm in diameter) filled with water to 21 cm (30 ± 1°C) and illuminated with 30 lux. Using a hand-held computer scoring system, a trained observer recorded the behavior of the mice for 6 min. These consisted of: (1) struggling that included forelimb movements breaking the water surface; (2) swimming where the mouse moves and fore- and hind-limbs do not break the surface of the water; and (3) floating, where the mouse makes small limb movements to stay above water without moving the trunk. The resulting data was analyzed using Observer 4.1 software (Noldus). Mice were dried with tissue after each trial and placed in a fresh cage atop a heating pad. Water was renewed before the next trial. Two middle-aged mice were excluded from the analysis due to inability to swim and three were excluded due to unannounced construction noise in the facility at the time point of analysis.

### Social Discrimination

Social Discrimination was assessed as previously described (Feil et al., 2009). The procedure consisted of two 4-min exposures of stimulus animals (ovariectomized 129Sv females) to the test animal in a fresh cage to which the test animal had been moved 2 h prior to testing. During the first exposure, one stimulus animal was exposed to the test animal. After a retention interval of 2 h, this stimulus animal was re-exposed to the test animal together with an additional, previously not presented stimulus animal. The duration of investigatory behavior of the test animal towards the stimulus animals was recorded by a trained observer with a hand-held computer. A social recognition index was calculated as time spent investigating the unfamiliar stimulus mouse/time spent investigating both the familiar and unfamiliar stimulus mouse.

### Tissue Preparation

Animals were deeply anesthetized using CO_2_ and perfused transcardially with 4% paraformaldehyde (PFA) in 0.1M phosphate buffer. Brains were dissected from the skulls, post-fixed overnight in 4% PFA at 4°C and then transferred to a 30% (w/v) sucrose solution until saturated. Brains from a subset of animals from each group were then sectioned on a dry ice-cooled block with a sliding microtome (Leica, Bensheim) into 40 μm-thick coronal free-floating sections and stored at −20°C in a cryoprotectant solution containing 25% ethylene glycol and 25% glycerine in phosphate buffer. A one-in-eight series of sections was taken for analysis from the brains of a subset of animals from each group.

### Immunostaining

For Ki67 staining, an avidin-biotin complex (Elite ABC kit, Vector, Burlingame, CA, USA) method was employed with a primary polyclonal rabbit anti-Ki67 antibody (1:200; NCL-Ki67p, Novacastra, Newcastle upon Tyne, UK), a secondary biotinylated goat anti-rabbit IgG (1:300, Jackson Immunoresearch Laboratories Inc., West Grove, PA, USA) and 3, 3^′^-diaminobenzidine (DAB) as the chromogen. Sections were counterstained with cresyl violet nissl stain (Sigma, Germany). For DCX immunostaining, an ABC protocol similar to that employed by Rao and Shetty (2004) was used. A primary goat polyclonal anti-DCX antibody (1:200, sc-8066, Santa Cruz Biotechnology, Santa Cruz, CA, USA) was used with a biotinylated rabbit anti-goat IgG (1:300; Jackson Immunoresearch Laboratories Inc.) and DAB as the chromogen.

### Quantification of Ki67+ and DCX+ Neurons

Estimation of the total number of DCX+ and Ki67+ cells was determined using unbiased stereology with the optical fractionator method and the semiautomatic StereoInvestigator system (MicroBrightField Inc., Williston, VT, USA). For DCX+ and Ki67+ cell estimation, the region of interest was traced in every 8^th^ section and the reference volume was determined. Immunopositive cells were quantified by systematic random sampling using the following settings: DCX+ cells in the granule cell layer of the hippocampal dentate gyrus and OB granule cell layer: a scan grid size of 200 × 200 μm and a counting frame of 100 × 100 μm; Ki67+ cells in the SGZ of the dentate gyrus: a scan grid size and counting frame size of 100 × 100 μm [an area sampling fraction of one is commonly used for counting rare cell populations, see (Latchney et al., 2014)]; Ki67+ cells in the SVZ: a scan grid size of 40 × 100 μm and a counting frame of 30 × 20 μm. Cells that intersected the uppermost focal plane or the lateral exclusion borders of the counting frame were not quantified. For each brain area the following number of sections were analyzed per animal: dentate gyrus = 7 sections; SVZ = 5 sections and OB = 5 sections.

### Statistical Analysis

Numerical analyses were performed using GraphPad Prism version 6.04 for Windows, GraphPad Software, La Jolla California USA, http://www.graphpad.com. For experiment 1, a two-way analysis of variance (ANOVA) was used with Bonferroni’s test corrected for multiple comparisons. For the behavioral analysis, we designated parameters that were significant after the Bonferroni’s correction for 11 indices as having “study-wide significance” and the term “nominal significance” was applied to parameters where there was a significance of *p* < 0.05 that did not surpass the Bonferroni’s corrected p value threshold. For experiment 2, Student’s *t*-tests were used to detect differences between the control and mutant mice. As no clear effects of gender were detected (see Table 3 for ANOVA result data), the data from male and female mice were pooled for these analyses.

## Results

### DCXCreER^T2^; DTA Mice as a Transgenic Model for the Selective Ablation of Newly Born Neurons in the Adult Brain

To assess the function of newly-born neurons in the adult brain, we generated an inducible double transgenic mouse model (DCXCreER^T2^; DTA mice; Figure 1A) where mice with CreER^T2^ under the control of the DCX promoter were crossed with mice with a lacZbpA-flox DTA cassette incorporated into the ubiquitously expressed ROSA26 promoter. Thus, DCX+ cells in these double transgenic mice were predisposed to undergo apoptotic cell death once TAM had bound to ER^T2^. The resulting activation of the Cre, with the ensuing removal of a floxed stop codon, thereby led to transcription of the DTA from the Rosa26 locus with subsequent cell death. As the control mice were single transgenic DCX CreER^T2^ mice without DTA, their DCX+ cells were not ablated by TAM administration.

Due to the potential for “leaky” effects of the CreER^T2^ system, we first performed an RT-PCR analysis of hippocampal DTA expression with and without the TAM treatment. With this analysis, we observed that there was only DTA expression in the Cre+ and DTA+ mice with TAM treatment and not without (Figure 1B). Having ascertained that there was minimal potential for non-specific effects of DTA activation, we proceeded with an experiment to assess the behavioral function of transiently DCX-expressing neurons in the adult brain (timeline for this experiment is illustrated in Figure 1C). Following a series of behavioral tests, we sacrificed the mice 2 days after the last assay. All results from the two way ANOVA analysis are shown in Table 1 and animal numbers for each test are shown in Table 2 in the Appendix.

**Table 1:** **Two-way ANOVA table with genotype and age as main factors**.

	Genotype			Age			Genotype × Age		
	*F*	*df*	*p*	*F*	*df*	*p*	*F*	*df*	*p*
**Open Field**
Distance moved (cm)	0.32	1, 29	0.58	0.01	1, 29	0.91	4.94	1, 29	0.03
Rearing (#)	1.33	1, 29	0.26	0.07	1, 29	0.79	2.55	1, 29	0.12
Center time (%)	0.35	1, 29	0.56	1.60	1, 29	0.22	1.19	1, 29	0.28
**Light/Dark Box**
Entries into light box (#)	0.28	1, 29	0.60	5.76	1, 29	0.02	1.46	1, 29	0.24
Time in light box (%)	0.74	1, 29	0.40	0.11	1, 29	0.74	0.16	1, 29	0.69
**Y Maze**
Spontaneous alternations (%)	0.08	1, 29	0.78	2.13	1, 29	0.16	0.64	1, 29	0.43
Alternate arm returns (%)	0.02	1, 29	0.89	3.78	1, 29	0.06	1.21	1, 29	0.28
**Social Discrimination**
Recognition index	5.91	1, 29	0.022	1.85	1, 29	0.18	0.04	1, 29	0.85
Investigation time (s)	0.17	1, 29	0.68	5.38	1, 29	0.03	0.34	1, 29	0.57
**Forced Swim Test**
Time floating (s)	44.47	1, 24	<0.0001	22.08	1, 24	<0.0001	3.03	1, 24	0.09
Time swimming (s)	29.95	1, 24	<0.0001	18.12	1, 24	0.0003	2.71	1, 24	0.11
**Histology**
DCX+ cells DG(#)	111.3	1, 13	<0.0001	67.76	1, 13	<0.0001	20.33	1, 13	0.0006
Ki67+ cells SGZ (#)	10.90	1, 11	0.007	13.66	1, 11	0.004	1.77	1, 11	0.21
DCX+ cells OB (#)	14.92	1, 12	0.002	11.60	1, 12	0.005	0.16	1, 12	0.70
Ki67+ cells SVZ (#)	0.52	1, 11	0.49	2.60	1, 11	0.14	0.01	1, 11	0.90

*Statistical table showing two-way ANOVA results for all analyses in Experiment 1 with age and genotype as main factors*.

**Table 2:** **Number of mice per group for each test**.

Age	3 months		10 months	
Genotype	Control	TG	Control	TG
Open Field	*N =* 3 m, 3 f	*N =* 4 m, 5 f	*N =* 3 m, 6 f	*N =* 3 m, 6 f
Light/Dark Box	*N =* 3 m, 3 f	*N =* 4 m, 5 f	*N =* 3 m, 6 f	*N =* 3 m, 6 f
Y Maze	*N =* 3 m, 3 f	*N =* 4 m, 5 f	*N =* 3 m, 6 f	*N =* 3 m, 6 f
Social Discrimination	*N =* 3 m, 3 f	*N =* 4 m, 5 f	*N =* 3 m, 6 f	*N =* 3 m, 6 f
Forced Swim Test	*N =* 3 m, 3 f	*N =* 4 m, 5 f	*N =* 3 m, 5 f	*N =* 3 m, 2 f
DCX+ cell counts	*N =* 3 m, 1 f	*N =* 2 m, 2 f	*N =* 4 m, 1 f	*N =* 3 m, 1 f
Ki67+ cell counts	*N =* 2 m, 1 f	*N =* 1 m, 2 f	*N =* 4 m, 1 f	*N =* 3 m, 1 f

*Table showing the number of animals per group for each analysis and genotype (Control and transgenic (Tg)) with details of breakdown for males (m) and females (f)*.

**Table 3:** **Two-way ANOVA table with genotype and sex as main factors**.

	Genotype			Sex			Genotype × Sex		
	*F*	*df*	*p*	*F*	*df*	*p*	*F*	*df*	*p*
**Open Field **
Distance moved (cm)	0.63	1, 29	0.43	2.49	1, 29	0.13	0.49	1, 29	0.49
Rearing (#)	1.11	1, 29	0.30	1.61	1, 29	0.21	0.69	1, 29	0.41
Center time (%)	0.002	1, 29	0.97	1.60	1, 29	0.22	4.06	1, 29	0.053
**Light/Dark Box**		
Entries into light box (#)	0.13	1, 29	0.72	0.10	1, 29	0.76	0.0006	1, 29	0.98
Time in light box (%)	1.13	1, 29	0.30	0.57	1, 29	0.46	0.71	1, 29	0.41
**Y Maze**
Spontaneous alternations (%)	0.18	1, 29	0.67	0.12	1, 29	0.73	0.07	1, 29	0.79
Alternate arm returns (%)	0.006	1, 29	0.94	0.49	1, 29	0.49	1.13	1, 29	0.30
**Social Discrimination**
Recognition index	5.09	1, 29	0.03	0.84	1, 29	0.37	0.01	1, 29	0.92
Investigation time (s)	0.29	1, 29	0.59	2.92	1, 29	0.10	0.52	1, 29	0.47
**Forced Swim Test**
Time floating (s)	31.75	1, 24	<0.0001	0.84	1, 24	0.37	0.48	1, 24	0.50
Time swimming (s)	23.66	1, 24	<0.0001	0.19	1, 24	0.66	0.31	1, 24	0.58
**Histology**
DCX+ cells DG(#)	10.55	1, 13	0.006	0.07	1, 13	0.80	0.02	1, 13	0.89
Ki67+ cells SGZ (#)	3.35	1, 11	0.09	0.28	1, 11	0.61	0.0001	1, 11	0.99
DCX+ cells OB (#)	2.70	1, 12	0.12	0.23	1, 12	0.64	0.10	1, 12	0.75
Ki67+ cells SVZ (#)	0.003	1, 11	0.96	1.44	1, 11	0.25	1.15	1, 11	0.31

*Statistical table showing two-way ANOVA results for all analyses in Experiment 1 with sex and genotype as main factors*.

**Figure 2:**
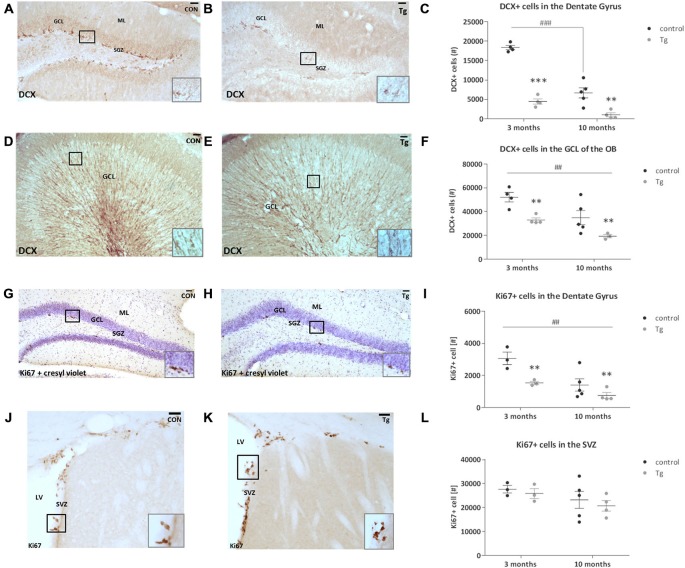
**Effect of transgene expression activation on number of DCX+ and Ki67+ cells in the subgranular zone (SGZ)/hippocampus and subventricular zone (SVZ)/olfactory bulb (OB) systems of DCXCreER^T2^; DTA mice. (A,B)** Representative photomicrographs of DCX+ cells in the hippocampal dentate gyrus of control (CON) and transgenic (Tg) mice and at higher magnification inset. **(C)** DCX+ cell number was reduced in the dentate gyrus of the transgenic mice compared to controls at both ages as well as in the 10 month old control mice compared to 3 month old controls. **(D,E)** Representative photomicrographs of DCX+ cells in the OB granular cell layer (GCL) and at higher magnification inset. **(F)** DCX+ cell number was reduced in the OB of the transgenic mice that were 3 and 10 months at time of DTA transgene activation. DCX+ cell number was decreased in the mice from the age of 10 months compared to those from the age of 3 months. **(G,H)** Representative photomicrographs of Ki67+ cells in the hippocampal SGZ counterstained with cresyl violet nissl stain. **(I)** The number of Ki67+ cells was reduced in the transgenic mice that were at both 3 and 10 months at the time of activation of transgene expression. The number of Ki67+ cells was reduced in the mice from 10 months of age compared to those from 3 months. **(J,K)** The number of Ki67+ cells in the SVZ. **(L)** There were no clear differences between the genotypes at either age. Scale bars, 40 μm, ML, Molecular layer; LV, Lateral ventricle. *N* = 3–5 per group. **p < 0.01, ***p < 0.001 vs. control mice. ^##^*p* < 0.01, ^###^*p* < 0.001 mice from 3 months vs. mice from 10 months. Data are means ± SEM.

To assess how effective this DCXCreER^T2^; DTA system was in ablating DCX+ cells in the brain at two different ages (from 3 and 10 months), using stereological approaches we quantified the number of DCX+ cells in the dentate gyrus of the hippocampus and the granular cell layer (GCL) of the OB (Figures 2A,B,D,E). DCX expression in the adult brain has also been shown to reflect overall levels of neurogenesis and DCX+ cell quantification can be used as an alternative to BrdU pulse/chase analysis (Couillard-Despres et al., 2005). Optical fractionator estimates showed that this DTA transgene expression resulted in a ~78% reduction in DCX+ cell number in the mice that were 3 months old at the time of induction (i.e., the age when the transgene was first activated through TAM administration; two way ANOVA Genotype × Age interaction effect: *F*_(1,13)_ = 20.33, *P* = 0.0006, Bonferroni’s test: *p* < 0.001, Figure 2C) and a 71% decrease in the mice that were 10 months old at induction (Bonferroni’s test: *p* < 0.01, Figure 2C). The control mice also undergo an age-related decrease in the number of DCX+ cells between 3 and 10 months (Bonferroni’s test: *p* < 0.001). In the GCL of the OB, there was a 33% decrease in DCX+ cell number in the mice that were 3 months at the time of induction and a 26% reduction in the mice that were 10 months (Two-way ANOVA main effect of Genotype: *F*_(1,12)_ = 14.92, *P* = 0.002, Figure 2F). There was also an age-dependent decrease in the number of DCX+ cells in the OB GCL between 3 and 10 months of age (Two-way ANOVA main effect of Age: *F*_(1,12)_ = 11.60, *P* = 0.005). As Ki67 represents both uncommitted Type 2a cells (Ki67+/DCX−) and neuronally-committed Type 2b/3 cells (Ki67+/DCX+; Kempermann et al., 2004), we quantified the number of Ki67+ proliferating cells in the SGZ of the hippocampal dentate gyrus and in the SVZ along the walls of the lateral ventricles (Figures 2G,H,J,K). The DTA expression resulted in a 50% and a 53% reduction in the number of proliferating cells in the SGZ of mice that were 3 and 10 months at the time point of induction, respectively (Figure 2I, Two-way ANOVA main effect of Genotype: *F*_(1,11)_ = 10.90, *P* = 0.007). There was also a significant age effect where the number of Ki67 positive cells in this region decreased with age (Two-way ANOVA main effect of Age: *F*_(1,11)_ = 13.66, *P* = 0.004). In the SVZ, there was no clear difference between the control and transgenic mice that were either 3 or 10 months at the time point of induction (Figure 2L).

### Selective Reduction of Immature Neurons Results in an Age-Independent Social Discrimination Deficit Without Clear Changes in Anxiety-Related Behavior

With this now established model that results in the selective decrease of newly-born neurons, we determined the function of these neurons in a series of different behavioral assays subsequent to DCX+ cell reduction. These tests covered aspects of locomotor activity, exploration, anxiety-related behavior, simple spatial working memory, short-term olfactory learning in a social context and stress reactivity/depression-related behavior.

**Figure 3:**
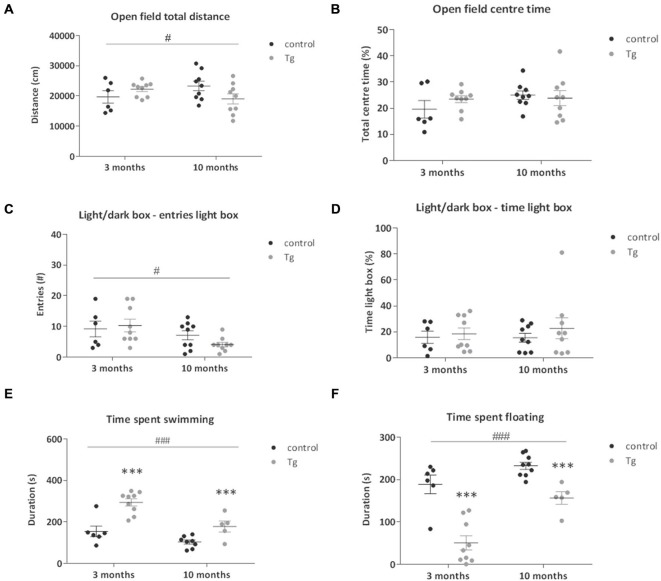
**Behavior tests of emotionality of DCXCreER^T2^; DTA mice. (A)** Total distance traveled and **(B)** total percentage time spent in the center of the Open Field in control and transgenic (Tg) mice. There was a small increase in total distance traveled in the transgenic mice from 3 months with the opposite effect in those from 10 months. ^#^*p* < 0.05 age × genotype interaction. *N* = 6–9 per group. **(C,D)** There were no clear differences between the genotypes at either age in the number of entries into or time spent in the lit compartment of the Light/Dark Box. Mice from 10 months of age made less light box entries compared to those from 3 months. *N* = 6–9 per group. **(E)** In the Forced Swim Test, the transgenic mice showed increased swimming activity at both ages tested and the mice from 10 months of age showed lower swimming activity compared to those from 3 months regardless of genotype. *N* = 5–9 per group. **(F)** Time spent immobile in the Forced Swim Test was decreased in the double transgenic mice compared to controls at both ages tested. There was an increase in the immobility time in the mice from 10 months of age compared to those from 3 months. *N* = 5–9 per group. ***p < 0.001 vs. control mice, ^#^*p* < 0.05, ^###^*p* < 0.001 mice from 3 months vs. mice from 10 months. Data are means ± SEM.

Previous studies have suggested a role for adult neurogenesis in anxiety-related behavior, however this evidence remains controversial (Reviewed by Petrik et al., 2012). We thus tested the DCXCreER^T2^; DTA mice in two tests of anxiety-related behavior starting 21 days after the first TAM injection. In the Open Field, a test of locomotor activity, exploration and anxiety-related behavior, we did not see a clear genotype effect on the anxiety-related index of % time spent in the central more aversive zone when DTA was induced at either 3 or 10 months of age (Figure 3B). Nevertheless, there was a significant interaction effect on locomotor activity (total distance traveled) in the Open Field. A slight increase was detected in the transgenic mice where DTA was induced at 3 months of age with the opposing effect in the transgenic mice induced at 10 months (Figure 3A, Two-way ANOVA interaction effect: *F*_(1,29)_ = 4.94, *P* = 0.03). This effect was not significant in *post hoc* testing. There were no significant genotype effects on exploratory/rearing activity in this environment (data not shown).

In the Light/Dark Box, another test of anxiety-related behavior that exploits a rodent’s natural aversion to open brightly lit spaces, there were no significant genotype effects on the anxiety indices of number of entries or the % time spent in the light box when DTA was activated at either 3 or 10 months of age (Figures 3C,D, Table 1). There was a nominally significant decrease in the number of entries into the light box by the mice from 10 months of age when compared to the mice from 3 months (Two-way ANOVA main effect of age: *F*_(1,29)_ = 5.76, *P* = 0.02).

The mice were tested for stress reactivity and depression-related behavior in the Forced Swim Test. An immobile posture assumed by the mouse in this inescapable situation is interpreted as a state of behavioral despair. During this 6 min test, we found that the amount of floating increases, while the amount of swimming decreases, with age in these mice (Two-way ANOVA main effect of Age: floating *F*_(1,24)_ = 22.08, *P* < 0.0001; swimming *F*_(1,24)_ = 18.12, *P* = 0.0003). We also observed that the transgenic mice showed a clear study-wide significant decrease in immobility/floating time (Two-way ANOVA main effect of Genotype: *F*_(1,24)_ = 44.47, *P* < 0.0001; Figure 3F) and increase in swimming time (Two-way ANOVA main effect of Genotype: *F*_(1,24)_ = 29.95, *P* < 0.0001; Figure 3E). There were no significant effects of transgene activation on struggling time in the Forced Swim Test (Data not shown).

**Figure 4:**
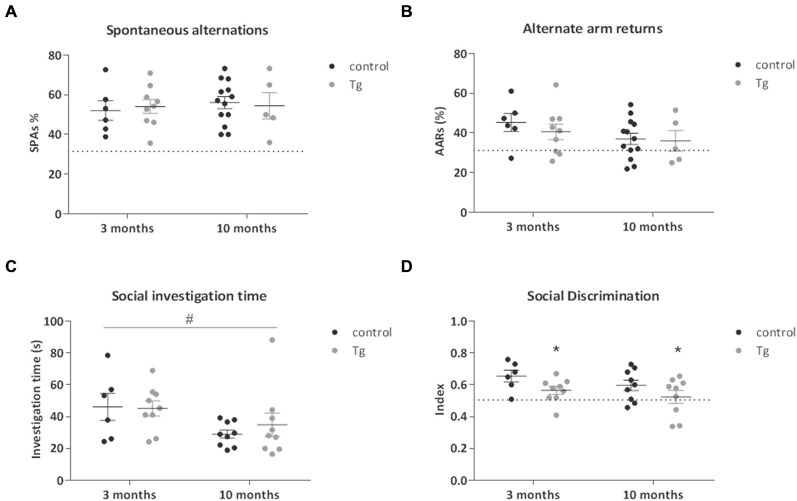
**Tests of cognition in DCXCreER^T2^; DTA mice. (A)** There were no clear effects of DCX+ cell deletion in the transgenic (Tg) mice compared to controls on either the number of spontaneous alternations or **(B)** the number of alternate arm returns in the Y Maze test of spatial working memory. Dotted line represents 33% chance level. **(C)** There was no clear effect of DCX+ cell ablation on the amount of time spent in social investigation during the habituation phase of the Social Discrimination Test. Social investigation was lower in the mice from 10 months of age compared to those from 3 months. **(D)** The social recognition index was lower in the transgenic mice compared to controls from both 3 and 10 months. Dotted line represents 50% chance level. *N* = 6–9 per group. *p < 0.05 vs. control mice, ^#^*p* < 0.05 mice from 3 months of age vs. mice from 10 months. Data are means ± SEM.

Given the previously established role played by hippocampal neurogenesis in spatial memory, we performed a simple test of spatial working memory; the Y Maze. There were no clear genotype-related effects detected on the total % spontaneous alternations or alternate arm returns in this test in mice where DTA was induced either at 3 or 10 months of age (Figures 4A,B, Table 1).

We next examined social behavior and social recognition ability in the Social Discrimination Test. This is a test of short-term olfactory learning and memory in a social context. There were no clear genotype effects on the social investigation time exhibited by the transgenic mice during the habituation phase of the procedure (Figure 4C). There was a nominally significant main effect of age on the level of social investigation, where the older mice spent less time engaged (Two-way ANOVA main effect of Age: *F*_(1,29)_ = 5.38, *P* = 0.03). Concerning the effects of genotype, the double transgenic mice showed a nominally significant decrease in social discrimination ability, seen as a reduced recognition index. This deficit was evident when DTA was activated at both 3 and 10 months of age (Figure 4D; Two way-ANOVA main effect of Genotype: *F*_(1,29)_ = 5.91, *P* = 0.022). As the uncorrected *p* value for this social discrimination effect did not reach study-wide significance, there was the possibility that this result could be a false positive and so replication was necessary. Thus, in a subset of the mice (*n* = 6 controls, *n* = 6 double transgenic mice) that were administered TAM in the reversal Experiment 2, we repeated the behavioral testing battery used in Experiment 1 while the DTA transgene was activated. We again observed a decreased recognition index in the double transgenic mice (*t*_(10)_ = 3.90, *p* = 0.003; Figure S1).

### Social Discrimination Deficit Could be Reversed by Repopulating the Neurogenic Zones with Newly-Born Neurons

**Figure 5:**
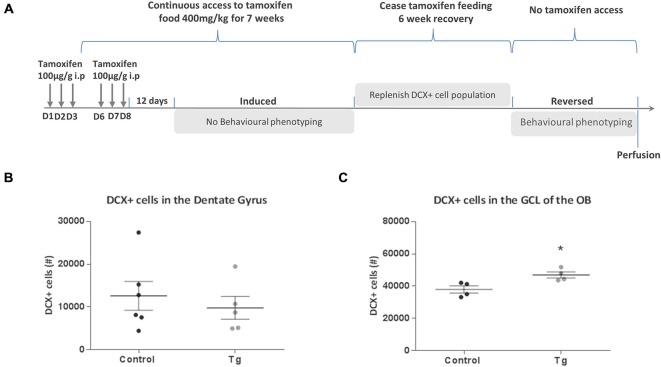
**Effect of repopulating the neurogenic zones with DCX+ cells in DCXCreER^T2^; DTA mice. (A)** Experimental timeline for Experiment 2. **(B)** No significant difference in the number of DCX+ cells in the hippocampal dentate gyrus between control and transgenic (Tg) mice without TAM dosing. **(C)** There was an increase in the number of DCX+ cells in the OB GCL of the double transgenic mice compared to control mice without TAM dosing. *N* = 4–6 per group. *p < 0.05 vs. control mice. D, day; ip, intraperitoneal; GCL, granular cell layer; OB, Olfactory bulb. Data are means ± SEM.

To determine whether the change in behavior in these mutant mice was due to loss of newly-born neurons, we wanted to see if it would be possible to reverse these effects by repopulating the neurogenic zones with newly-born neurons. The timeline of this reversal experiment is depicted in Figure 5A. An identical procedure to the first experiment was implemented where DTA was first activated with TAM administration, followed by continuous access to TAM-enriched food for 7 weeks (to correspond to the period necessary to test behavior of the animals before). Cessation of TAM-enriched food feeding then ensued for a period of 6 weeks and behavioral testing of the mice began. On completion of the behavioral analysis, mice were perfusion fixed and the number of DCX+ cells was quantified in the dentate gyrus of the hippocampus and the GCL of the OB. From this analysis, there were no significant differences in the number of DCX+ cells in the dentate gyrus between the groups (*t*_(9)_ = 0.63, n.s.; Figure 5B). There was now an increase in the number of DCX+ cells in the GCL of the OB (*t*_(6)_ = 3.12, *P* = 0.021; Figure 5C).

**Figure 6:**
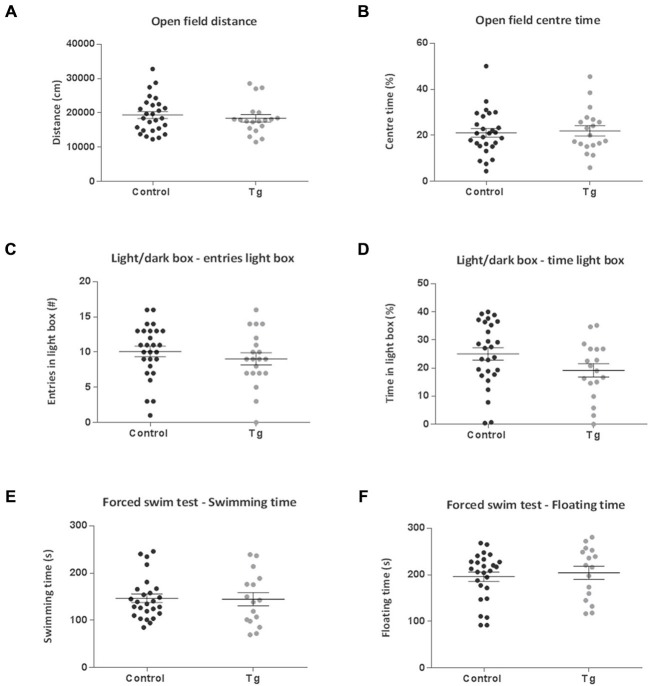
**Emotion-related behavior effects of repopulating the neurogenic zones with DCX+ cells in DCXCreER^T2^; DTA mice.** There were no differences between the control and transgenic (Tg) mice subsequent to a 6 week recovery period from tamoxifen administration in terms of **(A,B)**. Open Field total distance traveled or percentage time spent in the center **(C,D)** or in the number of entries into and time spent in the lit compartment of the Light/Dark Box. **(E,F)** Subsequent to repopulating the neurogenic zones with DCX+ cells there were no clear differences between the groups for either swimming or immobility time in the Forced Swim Test. *N* = 16–26 per group. Data are means ± SEM.

**Figure 7:**
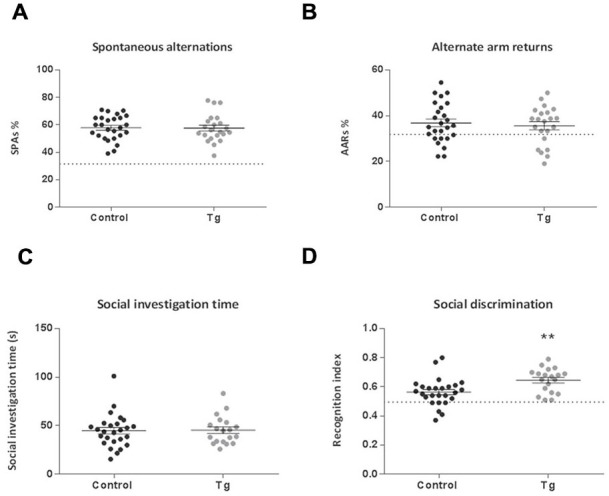
**Cognitive behavior effects of repopulating the neurogenic zones with DCX+ cells in DCXCreER^T2^; DTA mice.** There were no clear differences between transgenic (Tg) mice compared to controls subsequent to repopulating the adult neurogenic zones with DCX+ cells in either **(A)** the number of spontaneous alternations or **(B)** the number of alternate arm returns in the Y Maze test of spatial working memory. Dotted line represents 33% chance level. There were also no differences between the groups in terms of **(C)** social investigation time. **(D)** The social discrimination index was now significantly increased in the transgenic mice following the 6 week recovery period compared to controls. Dotted line represents 50% chance level. *N* = 19–26 per group. **p < 0.01 vs. control mice. Data are means ± SEM.

In the Open Field, Light/Dark Box and Y Maze test, there were no significant differences between the control and transgenic mice (Figures 6A–D, 7A,B). In terms of social discrimination memory, following a 6 week TAM washout period, there was now a significant increase in the recognition index of the double transgenic mice relative to the controls (*t*_(43)_ = 3.03, *P* = 0.004; Figure 7D) without significant differences in social investigation time (Figure 7C). In addition, there was no significant difference between the genotypes in terms of the immobility, struggling (Data not shown) or swimming time in the Forced Swim Test (Figures 6E,F).

## Discussion

Previous studies manipulating the level of adult neurogenesis have suggested a role for this process in hippocampal-dependent functions including spatial learning (Deng et al., 2009; Vukovic et al., 2013), pattern separation (Sahay et al., 2011) and emotion-related behavior (Revest et al., 2009), as well as in olfactory-related mating and maternal behaviors, but not simple odor discrimination or retention of odor-associated memory (Imayoshi et al., 2008; Lazarini and Lledo, 2011; Sakamoto et al., 2011). In spite of this volume of research, the precise contribution of adult neurogenesis to overall brain function is unclear. Thus, to further explore the functional importance of newly born neurons in the adult brain, we generated an inducible and reversible mouse model of adult neurogenesis reduction. Through the selective loss of DCX+ neurons we obviate potential side effects due to inhibition of other processes such as gliogenesis, and via inducibility, bypass potential developmental effects. We found reversible and age-independent behavioral effects on social memory and stress coping behavior with congruent changes in the number of DCX+ cells in the hippocampal dentate gyrus and OB GCL.

### The DCXCreER^T2^; DTA Mouse Enables Specific Reduction of Adult Neurogenesis

The induction of DTA expression resulted in a significant decrease in DCX+ cells in the hippocampal dentate gyrus; slightly more pronounced at 3 than 10 months of age. This ~78% reduction corresponds to the 77% recombination efficiency of the CreER^T2^ measured previously in fate mapping of the DCXCreER^T2^:CAG-CAT-EGFP reporter mice (Zhang et al., 2010). The remaining 22% DCX+ cells may either have escaped recombination induction or be delayed in cell death. The number of DCX+ cells in the GCL of the OB also decreased, in comparable magnitude at both ages of induction.

The number of proliferating Ki67+ cells significantly decreased in the SGZ, regardless of age at activation of the DTA transgene expression. As Ki67 represents both uncommitted Type 2a cells (Ki67+/DCX−) and neuronally committed Type 2b/3 cells (Ki67+/DCX+; Kempermann et al., 2004), we can deduce that there is a decrease in the latter of these cell types. We observed previously that a portion of EGFP+ cells (as an index of DCX+ cells) in the DCX CreER^T2^: CAG-CAT-EGFP mice were incorporating BrdU as proliferation marker (Zhang et al., 2010), but there was no co-localization between the EGFP signal and expression of GFAP (astrocyte marker), CNPase (oligodendrocyte marker) or Iba1 (microglial marker). This indicates that while the CreER^T2^ under DCX promoter control was found in cells still proliferating, these cells were neuronally committed and not involved in gliogenesis.

Our model is based on the human DCX promoter (shown to drive expression with a similar pattern to mouse endogenous DCX (Couillard-Despres et al., 2006; Zhang et al., 2010). Another recently generated DCX+ cell loss model (Vukovic et al., 2013) was a knock-in line in which a Venus-diphtheria toxin receptor (DTR) fusion sequence was controlled by the DCX promoter downstream of an internal ribosome entry site (IRES). A possible lower expression due to its position downstream of the IRES may account for differences between the models including that depletion of DCX+ cells occurred exclusively in the hippocampus and they did not observe differences in the number of proliferating (Ki67+) cells. The latter was surprising given the proportion of DCX-expressing cells that are still proliferating but may be explained by differences in the time point of analysis (they quantified Ki67 2d after an 11d DTA dosing regimen; Kempermann et al., 2004). Their model is therefore suited to assessing effects of ablation of immature neurons in the dentate gyrus, while our model is more amenable to longer-term analysis of combined hippocampal and OB neurogenesis reduction. This is of interest given the dearth of available genetic models addressing this issue.

### Loss of DCX+ Cells Leads to Changes in Emotionality

The outcome of the Open Field and Light/Dark Box tests suggests that, although there may be some subtle effects of the loss of newly-born neurons on exploratory behavior, there were no clear effects on anxiety-related behavior. The role played by adult neurogenesis in anxiety is controversial. While there were exceptions (Revest et al., 2009; Onksen et al., 2011), the consensus appears to be that without exposing mice with reduced neurogenesis to an additional challenge, there may not be changes in “baseline” levels of anxiety (Petrik et al., 2012; Groves et al., 2013), which is confirmed in the current study. Nevertheless, it should also be noted that a smaller sample size was used in this study that can decrease power to detect smaller differences in anxiety. In addition, DCX+ cells were quantified at the end of the behavioral analysis and thus it is not possible to quantify the DCX+ cell decrease at the time point of Open Field and Light/Dark box testing. However, we have previously established in a pilot study (data not shown) that there is already a significant (at least a 60%) decrease in DCX+ cells 26 days after the initial TAM injection, which approximately coincides with the time point of Open Field and Light/Dark Box testing in this study.

In the Forced Swim Test, we saw clearly (attained study-wide significance) decreased floating and increased swimming behavior. Most likely this reflects an altered coping response to this relatively more severe stressor, since there were no indications of increased activity or anxiety in these mice. Several neurogenesis studies did not reveal differences in this test (Holick et al., 2008; Bessa et al., 2009; Revest et al., 2009) with exceptions. It is not clear why there is variation but, as discussed previously (Petrik et al., 2012), this may be due to differences in the mode and extent of neurogenesis inhibition, timing and Forced Swim Test analysis used. For example, decreased immobility (scored during the last 4 min of the 6 min test) was detected in a constitutive cyclin D2 knock out mouse line where there was loss (90.4%) of adult hippocampal neurogenesis (Jaholkowski et al., 2009; Jedynak et al., 2014). Thus, the outcome of the current study reveals that an inducible loss of neurogenesis in adulthood produces a congruent effect. Another exception was in the GFAP-TK model of neurogenesis inhibition (Snyder et al., 2011), where loss of hippocampal neurogenesis (99%) was associated with increased immobility, but only during the first 2 min of the 6 min Forced Swim Test; there were no differences in the remainder. It was also shown that loss of hippocampal neurogenesis in the GFAP-TK mice led to hypersecretion of corticosterone in response to stress. Newly born neurons in the dentate gyrus may integrate novelty detection with activation of the hypothalamic-pituitary-adrenal axis and thereby modulate the stress response, assigning stress salience to a sensory context (Dranovsky and Leonardo, 2012).

### Social Discrimination Impairments were Contingent upon DCX+ Cell Loss

In the Y Maze, we did not observe any differences in the number of spontaneous alternations, indicating that loss of DCX+ cells does not produce deficits in this test. However, at both ages transgenic mice showed deficits in social discrimination memory, a measure of short-term memory in a social context depending on olfactory ability. This effect was replicated in an independent cohort of DCXCreER^T2^; DTA transgenic mice. There were no major differences in olfactory investigation time, suggesting the recognition difference is likely a true social memory deficit and not due to an inability to perform the test.

Functional evidence concerning the role of adult neurogenesis in olfactory-related behavior is conflicting. Aging was associated with a reduction of OB neurogenesis and odor discrimination in mice, but a genetic and an irradiation study found no effects of neurogenesis inhibition on odor discrimination ability (Enwere et al., 2004; Imayoshi et al., 2008; Lazarini et al., 2009). However, OB neurogenesis is necessary for olfactory learning and memory (Breton-Provencher et al., 2009; Arruda-Carvalho et al., 2014; Sakamoto et al., 2014) and mating behavior (Feierstein et al., 2010; Sakamoto et al., 2011). At variance with the only other genetic study to date that analyzed adult social recognition memory independent of mating behavior (Sakamoto et al., 2011), our results indicate an involvement of adult neurogenesis in social discrimination ability. Of note, while both hippocampal and OB neurogenesis were affected in the previous study, there were differences between the discrimination protocols. We exploited longer retention intervals and distinction between mice from a different strain. Inability of certain genetically manipulated mice to execute fine discrimination ability in a different strain may thus be of pertinence here (Macbeth et al., 2009). This is therefore the first evidence of a role for adult neurogenesis in this type of social discrimination memory.

### Ablation of DCX+ Cells is Reversible

Rescuing the effect of the transgene, by replenishing the neurogenic zones through cessation of the TAM treatment, is an important control. It excludes that the observed phenotype was due to loss of the rare non-proliferative extra-neurogenic DCX+ cells, potential leaky effects of the iCre or DTA technology causing DCX+ cell ablation during development or to random insertion sites of the DTA transgene or CreER^T2^ disrupting another endogenous gene. The latter control is essential as a DCXCreER^T2−^; R26lacZbpAfloxDTA^+^ control group was not included in the design of these experiments. Thus, analysis of the number of DCX+ cells in the hippocampal dentate gyrus after reversal revealed no significant differences. In the OB GCL, the number of DCX+ cells was now significantly increased in the transgenic mice, suggesting that chronic ablation of DCX+ cells in the SVZ/OB system leads to compensatory adjustments that precipitate an overshoot on reversal of the transgene expression. A quiescent state is perpetuated in neural stem cells through a combination of cell intrinsic and cell extrinsic factors including signaling of Notch, Wnt and Sox2 (Braun and Jessberger, 2014). Inhibition of the net level of neurogenesis, with its consequent decrease in new neuronal activity, could instigate the entry into the cell cycle of ordinarily quiescent progenitors, which continued to survive and migrate to the OBs when de novo transgene expression was silenced. Moreover, it is possible that the proliferation rates in the SVZ are higher than those in the SGZ and thus the recovery rate is greater on cessation of TAM treatment similar to that observed subsequent to ionizing radiation exposure (Hellstrom et al., 2009).

Behaviorally, we once again did not see any differences in the Open Field, Light/Dark box and Y Maze tests, but now the phenotype of decreased immobility/increased swimming was no longer visible in the Forced Swim test, suggesting that the effect is driven by the loss of DCX+ cells. Notably, in the Social Discrimination task, the transgenic mice now exhibited an increased recognition index, mirroring the concomitant increase in the number of DCX+ cells in the OB. Thus, the potential compensatory effect on increased SVZ neurogenesis may specifically underlie this enhanced social discrimination ability.

In conclusion, this is the first study to show that selective ablation of DCX+ neurons in the adult SGZ and SVZ leads to age-independent impaired social discrimination memory in mice, which, in the context of a former study (Sakamoto et al., 2011), appears to depend on the relative difficulty of the discrimination test applied. Loss of DCX+ neurons was associated with age-independent altered stress coping without a concomitant change in anxiety-related behavior, tallying with previous assertions that adult neurogenesis is necessary to buffer responses to more severe stress exposure. The reversal of these effects demonstrated that they were due to specific loss of DCX+ cells and the phenotype could be rescued by repopulating the adult neurogenic zones with new neurons. Overall, these results support the notion that adult neurogenesis plays a role in social memory and in stress coping but not necessarily in anxiety-related behavior.

## Author Contributions

LG, JZ, VGD, HF, MHDA, DVW, WW and SMH conceived this work; AZ contributed substantially to the design of the study and to the interpretation of the data. LG, JZ and KMN acquired and analyzed the data; LG, JZ and SMH interpreted the data and drafted the manuscript. All authors critically revised the manuscript, approved the final version to be published and agreed to be accountable for all aspects of the work in ensuring that questions related to its accuracy are appropriately investigated and resolved.

## Conflict of Interest Statement

The authors declare that the research was conducted in the absence of any commercial or financial relationships that could be construed as a potential conflict of interest.

## Abbreviations

SGZ: Subgranular zone
SVZ: Subventricular zone
RMS: Rostral migratory stream
TAM: Tamoxifen
DCX: Doublecortin
DTA: Diphtheria toxin A
GCL: Granular cell layer
OB: Olfactory bulb
Tg: Transgenic
ML: Molecular layer
LV: Lateral ventricle.
