# Supplementary figures and images for: Conditional Reduction of Adult Born Doublecortin-Positive Neurons Reversibly Impairs Selective Behaviors
Source: Front Behav Neurosci. 2015 Nov 12;9:302. doi: 10.3389/fnbeh.2015.00302 (PMC4642364; doi:10.3389/fnbeh.2015.00302)

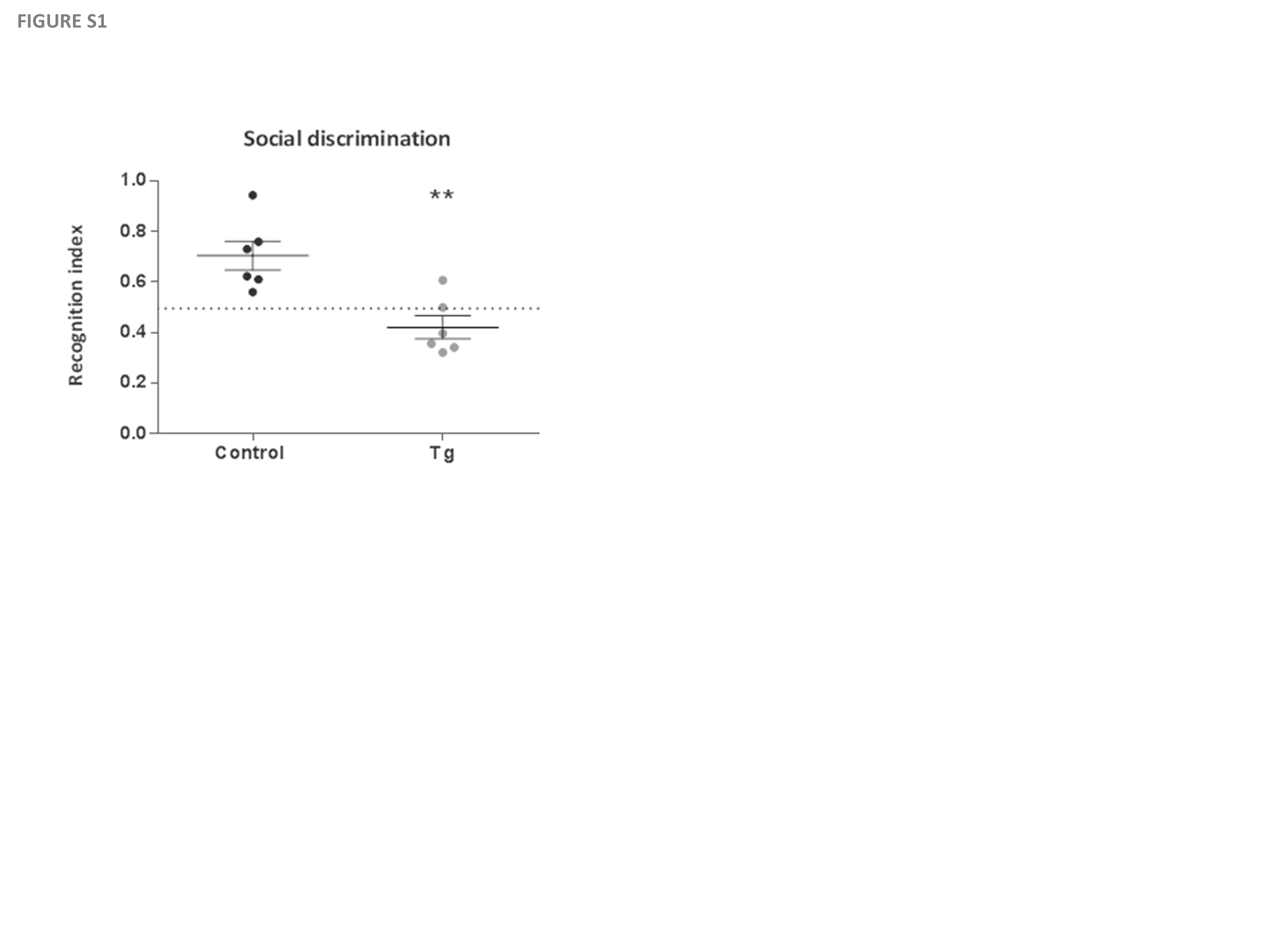

Supplement: Supplementary file 1 [file Image_1.tif]
